# Excessive Oxalic Acid Secreted by *Sparassis latifolia* Inhibits the Growth of Mycelia during Its Saprophytic Process

**DOI:** 10.3390/cells11152423

**Published:** 2022-08-05

**Authors:** Lili Shu, Miaoyue Wang, Shuang Wang, Yu Li, Hui Xu, Zhiheng Qiu, Tianlai Li

**Affiliations:** 1 College of Horticulture, Shenyang Agricultural University, Shenyang 110866, China; 2 Key Laboratory of Protected Horticulture of Education Ministry and Liaoning Province, Shenyang 110866, China; 3 Engineering Research Center of Chinese Ministry of Education for Edible and Medicinal Fungi, Jilin Agricultural University, Changchun 130118, China

**Keywords:** *Sparassis latifolia*, oxalic acid, mycelial growth, saprophytic process, the degradtion of lignocellulose, cultivation cycle

## Abstract

*Sparassis latifolia* is an edible and medicinal mushroom in Asia commercially cultivated on substrates containing pine sawdust. Its slow mycelial growth rate greatly increases the cultivation cycle. In this study, we mainly studied the role of oxalic acid (OA) secreted by *S. latifolia* in its saprophytic process. Our results show that crystals observed on the mycelial surface contained calcium oxalate monohydrate (COM) and calcium oxalate dihydrate (COD) according to X-ray diffraction (XRD). Vegetative mycelia secreted large amounts of OA during extended culture periods. However, high concentrations of OA decreased the mycelial growth rate significantly. Moreover, the degradation of lignocellulose was significantly inhibited under high concentrations of OA. These changes could be attributed to the significantly decreased activities of lignocellulose-degrading enzymes. In conclusion, by establishing a link between OA secretion by the mycelium and the slow growth rate of its saprophytic process, this work provides fundamental information for shortening the cultivation cycle of *S. latifolia*.

## 1. Introduction

*Sparassis latifolia*, also known as cauliflower mushroom, is a higher fungus with important nutritional and medicinal value. Being rich in flavor compounds, it is regarded as a good dietary supplement [1]. Moreover, as it contains numerous bioactive ingredients, its scientific and medical relevance has attracted attention in Korea, China, Japan, Germany, and the USA [2,3]. However, compared to other fungal cultures, its slow spawn running rate and long growth cycle pose major challenges in the industrial production of *S. latifolia*. Progress on the domestication, cultivation, and nutrient metabolism of *S. latifolia* has been slow, severely restricting its full-scale commercialization [4]. In particular, key factors restricting the growth rate of *S. latifolia* are poorly understood.

Our previous study found that the physiological characteristics of *S. latifolia* were very different from those of other fungal crops. It is not only an edible and medicinal fungus that can be cultured artificially, but also a pathogenic fungus that can cause basal rot in *Pinus koraiensis* [5]. Thus, *S. latifolia* shows a facultative pathogenic behavior. Because of this feeding pattern, research findings on conventional fungal crops cannot be used to guide its domestication or cultivation. The difficulty of providing optimal nutrition to *S. latifolia* cannot be easily resolved. This leads to stagnation in developing the commercial cultivation of this fungal crop [6,7].

Oxalate secretion by some parasitic fungi is known to be associated with fungal pathogenesis. OA plays an important role in helping plant pathogens to acquire nutrients, compete with other microorganisms in the environment, and regulate toxicity and pathogenicity [8]. In the early pathogenic stage, the accumulation of OA in the infected parts of host tissue leads to a decrease in pH, which generally falls to about 4.5 [9]. Several plant pathogenic fungi are unique in their ability to degrade lignocellulose due to the production of the relevant lignocellulose-degrading enzymes, such as manganese peroxidase (MnP), laccase, cellulase, amylase, and pectinase. These enzymes contribute not only to nutrient absorption, but also to the penetration, infection, colonization, and decay of host tissue. As the optimal pH value of these enzymes is usually lower than 5.0, the presence of OA provides an optimally acidic environment for these enzymes [10].

Furthermore, OA also plays a very important role in degrading wood. Many brown-rot and white-rot basidiomycetes produce large quantities of oxalate, such as *Tyromyces palustris* and *Ganoderma applanatum* [11]. OA is formed as a product of glucose metabolism when the hydrolysis of oxaloacetate is catalyzed by acetylhydrolase (OAH) [12]. Wood-degrading fungi have the ability to regulate extracellular OA levels. For example, *Postia placenta* may produce less OA to maintain the activity of lignocellulose-degrading enzymes [13]. OA has a dual effect on the Fenton reaction, which removes insoluble organic matter. OA can scavenge hydroxyl radicals, which promotes the Fenton reaction at low concentrations, but inhibits it as the concentration increases [14]. The degradation of pine sawdust, used in the cultivation of fungal crops, requires the participation of the Fenton reaction. Therefore, understanding this bidirectional regulatory effect of OA may help in finding optimal conditions for cultivating fungal crops.

At present, there is no explanation for the slow degradation of pine sawdust by *S. latifolia*. If this problem cannot be resolved, it will continue to restrict further developments in the utilization of *S. latifolia*. Although the production of OA appears to be widespread in fungi, the function of OA still remains to be fully understood [15]. Moreover, the relevance of OA secreted by *S. latifolia* in its saprophytic process has not been previously studied. The aim of this paper was to establish the role of OA secreted by *S. latifolia* in its saprophytic process.

## 2. Materials and Methods

### 2.1. Strain, Media, and Culture Conditions

The *S. latifolia* strain (No. CCMJ 1100) was provided by the Culture Collection Center of Mycology of Jilin Agriculture University. To obtain a homogenous pure culture of *S. latifolia*, a 5 mm-diameter punch from solid medium was inoculated onto the center of solid potato dextrose peptone agar (PDPA) and maintained at 24 °C [16]. To grow mycelia using submerged cultivation, ten pieces of 5 mm *S. latifolia* mycelial disc were inoculated into 250 mL Erlenmeyer flasks containing 100 mL of potato dextrose peptone broth (PDPB) and incubated in a rotary shaker (ZQLY-180F, Shanghai Zhichu Instrument Company Limited, Shanghai, China) at 24 °C, 150 rpm, for 10 days. To grow mycelia in pine sawdust medium (PSM), ten pieces of 5 mm *S. latifolia* mycelial disc were inoculated on the top of PSM in the bag (17 × 35 cm) and incubated at 24 °C. The PSM contained 76% pine sawdust, 18% wheat bran, 2% corn flour, 1.5% sucrose, 1.5% gypsum, and 1% calcium superphosphate. Each bag contained 1 kg of PSM with 65% humidity.

### 2.2. Microscopic Observations of the Mycelium and the Crystals Formed on Its Surface

To investigate the crystals forming on the surface of *S. latifolia* mycelia after prolonged incubation, a 5 mm mycelial disc was grown on a thin layer of PDPA on the microscope slides. After 30 days of incubation at 24 °C, the mycelium and crystals forming on the surface were observed under 3D super-depth digital microscopy (Zeiss Smartzoom 5, Carl Zeiss, Jena, Germany) and an optical microscope (ZEISS Primostar 3, Carl Zeiss, Jena, Germany). Moreover, to observe ultrastructural features of mycelia and the crystals, scanning electron microscopy (SEM, Inspect S50, FEI, Hillsboro, OR, USA) and transmission electron microscopy (TEM, Zeiss EVO18, Carl Zeiss, Jena, Germany) were also performed. To collect mycelial samples with crystals, *S. latifolia* mycelial discs were inoculated into the center of PDPA plates. After 20 days of incubation at 24 °C, glass slides were inserted obliquely into the medium at the edge of the colony. The plates were cultured at 24 °C for another 20 days. At this time point, glass slides containing mycelia and crystals were selected for observation. Samples for SEM and TEM were prepared according to a previously described method [17].

### 2.3. Identifying Crystal Components Using XRD

In order to analyze the components of the crystals formed on the surface of *S. latifolia* mycelia, crystal samples were detected with a Bruker D8 Venture diffractometer (Germany) using a previously established method with minor modifications [18]. Crystals were collected from the PDPA plates of *S. latifolia* after 40 days of incubation. The power XRD measurements were performed with graphite-monochromated Mo-Kα radiation (λ = 0.71073 Å) at a scanning rate of 2°/min and a scanning range (2*θ*) from 4° to 60°, voltage at 30 kV; 15 mA current, 0.5 s time constant, and 1.0 s counting time. The structure was solved with direct methods using Olex2 software (Olex2-1.5, https://www.olexsys.org/olex2/docs/getting-started/installing-olex2/, accessed on 28 April 2022) with the SHELXS structure solution program. The detected structural model was further refined using full-matrix least-squares on F2 with SHELXL-97 [19].

### 2.4. Quantifying OA Secretion by S. latifolia

Quantitative analysis of OA secreted by *S. latifolia* in culture was carried out using high-pressure liquid chromatography (HPLC) (Agilent 1260 Infinity II Prime, Agilent, Santa Clara, CA, USA) [20]. *S. latifolia* mycelial discs were placed in the center of a sterile cellophane sheet overlayed on top of PDPA plates and incubated at 24 °C. This set up allowed the diffusion of OA into the medium below. After 10, 15, 20, 25, 30, 35, or 40 days of incubation, the cellophane was lifted, and 0.5 g of the medium near the inoculation point was removed, placed into a centrifuge tube, and mashed. To extract OA, 8 mL of 2% sulfuric acid was added to the content of the tube and extracted at 25 °C, 170 rpm, for 2 h. Subsequently, the supernatant was collected via centrifugation (Sigma 3K30, Osterode, Germany) at 14,000 rpm for 10 min and filtered with sterile syringe filters (0.22 μm). Prior to quantitative measurements, a standard curve of OA (Sigma-Aldrich, St. Louis, MO, USA) was generated using a 100 μg/mL solution as the chemical reference substance. For the OA separation, a ZORBAX SB-Aq column (4.6 × 250 mm, 5 μm, Agilent, Santa Clara, CA, USA) was used. Mobile phase A included 2 mM sulfuric acid, pH1.96, and phase B was pure methanol, at a 9:1 ratio. The flow rate was 0.5 mL/min, and the column was maintained at 30 °C. Peak detection was performed with an ultraviolet (UV) detector (UV752N, Yoke Instruments Co., Ltd., Shanghai, China) at 210 nm. Experiments were performed in triplicate.

### 2.5. The Effects of Different OA Concentrations on Mycelial Growth of S. latifolia

To investigate the effects of OA on the mycelial growth of *S. latifolia*, PDPA media containing different concentrations of exogenous sterile OA were prepared. Other plates contained an inhibitor of OA synthesis 3,3-difluorooxaloacetate, prepared by a previously described method [21]. The concentration of 3,3-Difluorooxaloacetate was 98%. Every plate contained the same amount (10 mL) of media. For the sake of simplicity and brevity, for the rest of this paper, media containing 3,3-difluorooxaloacetate will be referred to as OA^-^. *S. latifolia* mycelial discs (5 mm) were inoculated onto the center of these modified PDPA plates and incubated at 24 °C. In addition, PSM supplemented with 10 mM OA or 4 mM OA^-^ were also used to investigate mycelial growth. Ten pieces of 5 mm mycelial discs were separately inoculated and incubated at 24 °C for 25 days. Experiments were performed in triplicate.

### 2.6. Observation of the Structure of Pine Sawdust under SEM

To establish the effects of OA on the degradation of pine sawdust by *S. latifolia*, SEM was used to analyze the morphology of pine sawdust. Ten pieces of 5 mm mycelial discs were inoculated into PSM supplemented with either 10 mM OA or 4 mM OA^-^. At the same time, ten pieces of 5 mm mycelial discs were inoculated into PSM. Pine sawdust samples were collected from the top part of these cultures after incubation at 24 °C for 40 days. Samples for SEM analysis were prepared according to a method described previously [22].

### 2.7. Determination of Cellulose, Hemicellulose, and Lignin Content of Pine Sawdust

After culturing *S. latifolia* on PSM (CK) and PSM supplemented with 10 mM OA (OA) and 4 mM OA^-^ (OA^-^) for 40 days at 24 °C, pine sawdust was collected, and its cellulose, hemicellulose and lignin content was determined. Cellulose, hemicellulose, and lignin content was measured using a previous method [23].

### 2.8. Effects of OA on Activities of Extracellular Degradation Enzymes of S. latifolia

To investigate the effect of different concentrations of OA on the activity of extracellular enzymes produced by *S. latifolia*, the fungus was cultured in 100 mL of PDPB media containing 5% pine sawdust. The liquid medium contained either OA at 10 mM, or OA^-^ at a final concentration of 4 mM. Ten *S. latifolia* mycelial discs (5 mm) were inoculated into the three versions of the media (CK: normal media, OA: media supplemented with 10 mM OA, OA^-^: media supplemented with 4 mM OA^-^). Subsequently, the flasks were transferred to a rotary shaker and incubated at 24 °C, 150 rpm, for 20 days. Liquid culture was collected via centrifugation (Sigma 3K30) at 6000× *g* for 10 min, and the supernatants were used as crude enzyme solutions. Samples were stored at −80 °C until assayed. All measurements were performed in triplicate.

Laccase activity was measured according to a previously described method, monitoring the oxidation of 2,2′-azinodi-3-ethyl-benzothiazoline-6-sulfuric acid (ABTS) (Sigma-Aldrich, St. Louis, MO, USA) [24]. A 300 µL aliquot of crude enzyme solution was incubated with 2.7 mL of 1 mM ABTS (in 50 mM sodium acetate buffer, pH4.5) for 5 min at 30 °C. One unit (U) of enzyme activity was defined as the amount of laccase required to oxidize 1 µmol ABTS (ε420 = 3.6 × 104 M^−1^∙cm^−1^) per min. MnP activity was measured as previously described [25]. Carboxymethyl cellulase (CMCase) was assayed as previously described [26], and hemicellulase was measured as previously described [27]. Amylase and pectinase activity was measured according to the method developed by Bedade et al. [28].

### 2.9. Statistical Analysis

Data are presented as the mean and the standard deviation (SD). Analysis of variance (ANOVA) and Duncan’s multiple range tests (*p* ≤ 0.05) were applied for data analysis. SPSS version 20.0 software (SPSS Inc., Chicago, IL, USA) was used for statistical analyses.

## 3. Results

### 3.1. Observation of Crystalline Characterizations under Different Microscopic Magnifications

**Figure 1:**
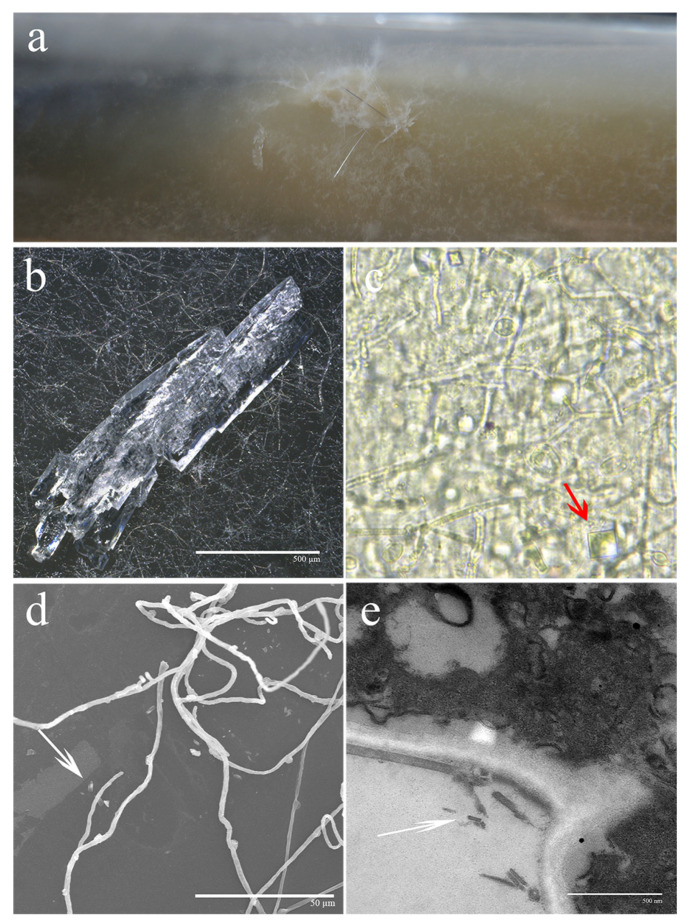
Morphological characterization of mycelium and crystals. The macroscopic morphology and microstructures were observed simultaneously using (**a**) a digital camera, (**b**) 3D digital microscopy, (**c**) light microscopy, (**d**) SEM, and (**e**) TEM. Crystals are indicated with red and white arrowheads.

After 30 days of incubation, visible, white, transparent crystals formed on the surface of *S. latifolia* mycelia (Figure 1a). When observed using a 3D digital microscopy, mycelia were evenly distributed and dense. On the surface of mycelia, complex crystalline structures could be seen, mostly comprising needle-like and irregular columnar structures (Figure 1b). Very regular square crystals could be observed at the red arrowhead using light microscopy (Figure 1c). SEM and TEM analyses allowed for the visualization of detailed crystal morphology. The ultrastructural morphology could be clearly characterized by using SEM and TEM. Regular rod-shaped and columnar crystals are shown by the white arrowheads (Figure 1d,e). The above-mentioned results together indicate that *S. latifolia* forms distinct crystals on the surface of mycelia after a prolonged incubation period.

### 3.2. XRD Analysis of Crystals

**Figure 2:**
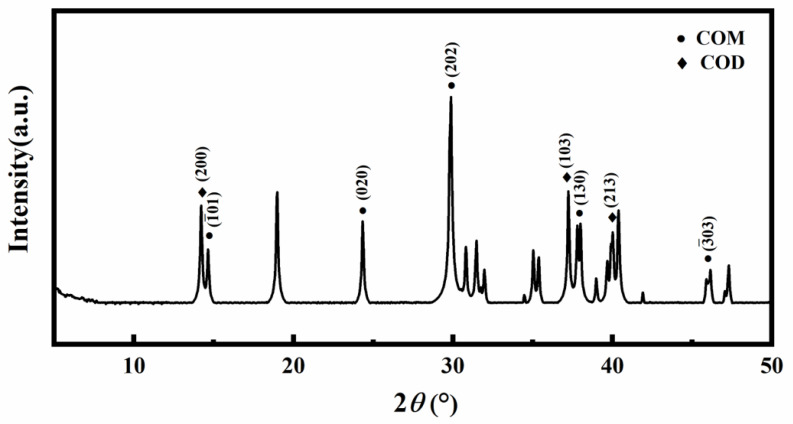
XRD diffraction diagrams of crystals. ●: COM, ◆: COD.

Based on XRD patterns, the specific composition of a crystal could be clearly established. Clear diffraction peaks could be observed at different degrees. Compared to standard COM, the crystals produced by *S. latifolia* produced several peaks that matched perfectly with typical peak positions of COM, such as ($\bar{1}$01), (020), (130), and ($\bar{3}$03) (Figure 2). Additional peaks appearing at (200), (103), and (213) were indexed as COD crystals. These results indicate that the crystals forming on the surface of *S. latifolia* mycelia were mainly composed of COM and COD.

### 3.3. S. latifolia Mycelia Secreted Large Amounts of OA during Longer Cultivation

**Figure 3:**
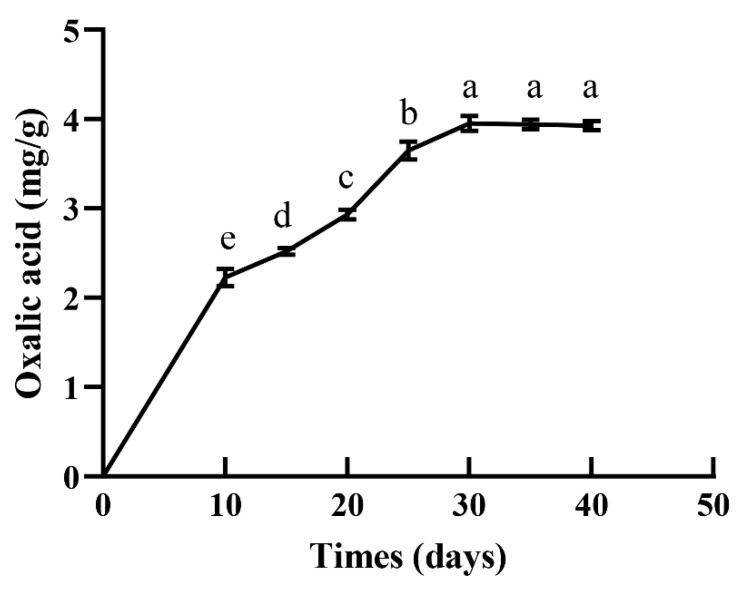
OA content in the growth medium at different time points during cultivation. Data were analyzed with Duncan’s ANOVA test. Error bars represent the standard deviation of three replicates. Different letters indicate significant differences between the lines (*p* ≤ 0.05).

The amount of OA secreted by *S. latifolia* into the growth medium was determined via HPLC. As shown in Figure 3, OA content increased gradually with the passage of cultivation time (Figure 3). It increased significantly from day 10 to day 25, reaching 3.65 mg/g in the medium by day 25. After this time point, the OA content of the medium did not significantly increase, but remained stable. These results indicate that *S. latifolia* secretes considerable amounts of OA during incubation.

### 3.4. The Level of OA Affected the Growth of Mycelia in Different Media

**Figure 4:**
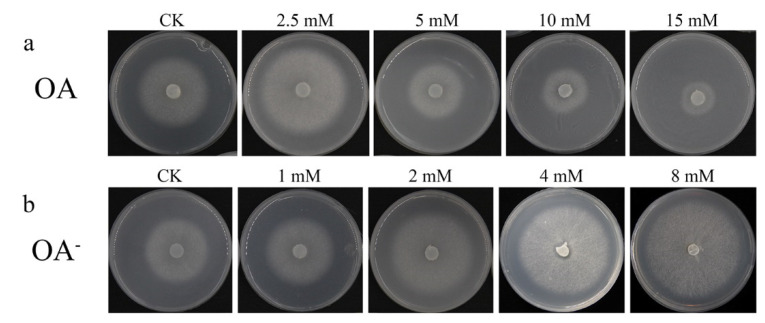
Growth of the *S. latifolia* mycelium on PDPA. (**a**) Growth of the *S. latifolia* mycelium on PDPA supplemented with different concentrations of OA; (**b**) growth of the *S. latifolia* mycelium on PDPA supplemented with different concentrations of OA^-^.

To determine the effect of OA on the mycelial growth of *S. latifolia*, mycelia were grown on a PDPA medium containing different concentrations of OA. Under these conditions, colony diameter was proportional to the rate of growth of *S. latifolia* mycelia. Compared to colonies grown on control PDPA media (CK), not containing any exogenous OA, the growth rate of mycelia in the presence of 2.5 mM OA was notably faster. However, as OA concentration increased further, from 5 mM to 15 mM, colony diameter decreased significantly (Figure 4a). The growth of mycelia was strongly inhibited in the medium with 10 mM and 15 mM OA. These results reveal that high concentration of OA could inhibit the mycelial growth of *S. latifolia*. On the other hand, compared with the PDPA medium (CK), growing mycelia on the OA^-^ medium resulted in increased colony sizes, which was dose-dependent on the concentration of the OA synthesis inhibitor in the OA^-^ medium (Figure 4b). Larger colony diameters were seen on OA^-^ plates containing 4 mM and 8 mM of OA^-^. These results further illustrate the inhibitory effect of high concentrations of OA on the mycelial growth of *S. latifolia*.

**Figure 5:**
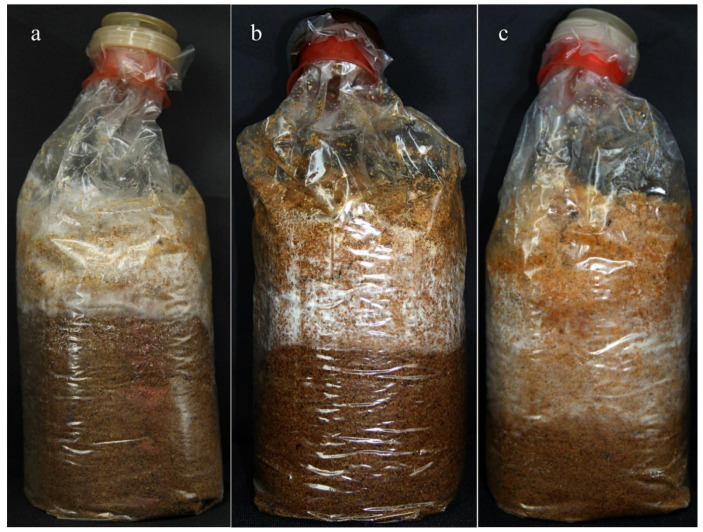
Growth of the *S. latifolia* mycelium in PSM. (**a**) Growth of the *S. latifolia* mycelium in PSM supplemented with 10 mM OA; (**b**) growth of the *S. latifolia* mycelium in PSM; (**c**) growth of the *S. latifolia* mycelium in PSM supplemented with 4 mM OA^-^.

**Figure 6:**
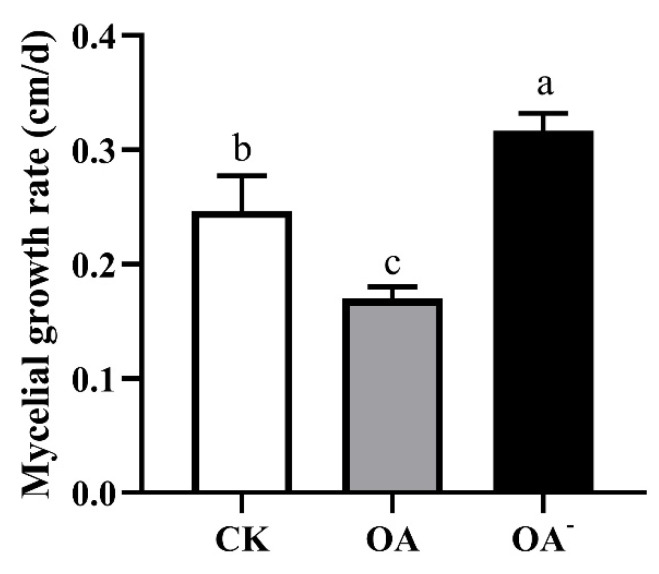
Growth rate of the *S. latifolia* mycelium in PSM supplemented with OA and OA^-^. CK: PSM, OA: PSM supplemented with 10 mM OA, OA^-^: PSM supplemented with 4 mM OA^-^. Data were analyzed with Duncan’s ANOVA test. Error bars represent the standard deviation of three replicates. Different letters indicate significant differences between the lines (*p* ≤ 0.05).

The growth of mycelia was also observed using the PSM supplemented with 10 mM OA or 4 mM OA^-^. These experiments produced similar results (Figure 5). The growth of the mycelium was significantly (*p* ≤ 0.05) inhibited in the PSM supplemented with 10 mM OA, with the mycelial growth rate being the slowest, only 0.32 cm/d (Figure 6). In contrast, mycelia on the OA^-^ PSM showed a significantly faster growth rate than that in CK or in the presence of OA. These comparisons amply illustrate that OA affected mycelial growth of *S. latifolia*, with excessive OA inhibiting growth.

### 3.5. High Concentrations of OA Inhibited the Degradation of Pine Sawdust by S. latifolia

**Figure 7:**
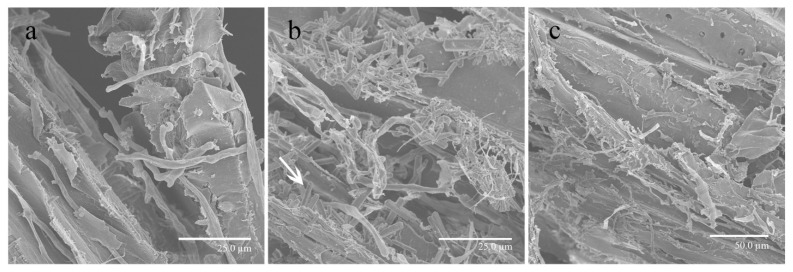
Distinguishing features of pine sawdust degraded by *S. latifolia*. (**a**) Pine sawdust collected from PSM; (**b**) pine sawdust collected from PSM supplemented with 10 mM OA, and mostly intact fibers; some bound macrofibrils and large numbers of crystals can be seen; (**c**) pine sawdust collected from PSM supplemented with 4 mM OA^-^. Crystal is indicated with white arrowhead.

SEM was used to qualitatively assess structural information regarding the impact of OA concentrations on the degradation of pine sawdust by *S. latifolia*. Under different OA concentrations, the ability of *S. latifolia* to degrade pine sawdust showed obvious differences. OA concentration significantly affected the structure of pine sawdust showing the destruction of the plant cell walls. Untreated pine sawdust showed structural changes with a slightly rougher surface, which is indicative of lignocellulose degradation by *S. latifolia* (Figure 7a). However, the degradation of pine sawdust was significantly inhibited at high concentrations of OA (10 mM). The fiber structure remained intact, and the surface of pine sawdust was very smooth (Figure 7b). Moreover, after the addition of excessive OA, a large number of crystals will be formed on the pine sawdust, such as the crystal indicated by the arrowhead (Figure 7b). Nonetheless, exposed macrofibrils and microfibrils were conspicuously visible in the pine sawdust supplemented with 4 mM OA^-^ (Figure 7c). In the presence of OA^-^, the surface of pine sawdust was very rough, indicating that it was strongly degraded by *S. latifolia*. Taken together, the above results suggest that a high concentration of OA was not conducive to the degradation of pine sawdust by *S. latifolia*.

### 3.6. High Concentration of OA Inhibited the Degradation of Lignocellulose by S. latifolia

**Figure 8:**
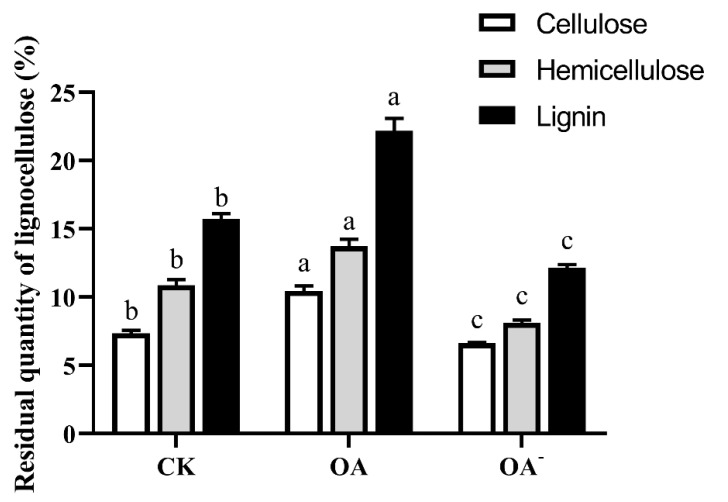
Residual quantity of lignocellulose in pine sawdust under different treatments after degradation of *S. latifolia*. Data were analyzed with Duncan’s ANOVA test. Error bars represent the standard deviation of three replicates. Different letters indicate significant differences between the columns (*p ≤* 0.05).

Under the same culture conditions, the residues of lignin, cellulose, and hemicellulose in pine sawdust directly represented the activity of the extracellular enzymes of *S. latifolia* on pine sawdust. The effects of OA concentrations on the degradation of cellulose, hemicellulose, and lignin were consistent. Under high OA concentrations, the degradation of all three components was significantly (*p* ≤ 0.05) inhibited (Figure 8). In the presence of OA^-^, the degradation of lignocellulose by *S. latifolia* improved greatly compared to the other two treatments. These results are consistent with the observations made by SEM, showing that high concentrations of OA inhibited the degradation of lignocellulose by *S. latifolia*.

### 3.7. High Concentration of OA Inhibited the Activity of Lignocellulose-Degrading Enzymes of S. latifolia

**Figure 9:**
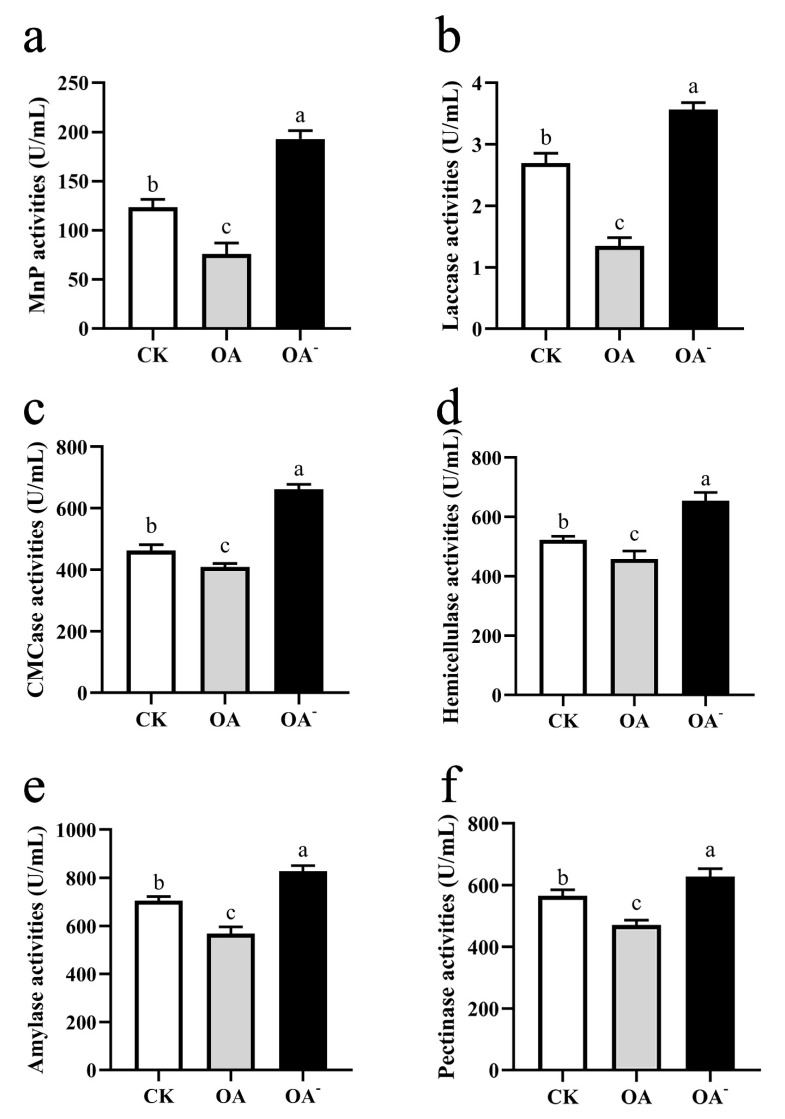
Activities of lignocellulose-degrading enzymes secreted by *S. latifolia*. Effects of different treatments (CK, OA, OA^-^) on activities of (**a**) MnP, (**b**) laccase, (**c**) CMCase, (**d**) hemicellulase, (**e**) amylase, and (**f**) pectinase. Data were analyzed with Duncan’s ANOVA test. Error bars represent the standard deviation of three replicates. Different letters indicate significant differences between the lines (*p* ≤ 0.05).

The activity of lignocellulose-degrading enzymes secreted by *S. latifolia* was closely related to its ability to degrade pine sawdust, affecting the growth rate of mycelia. Under high concentrations of OA, the extracellular lignocellulose-degrading enzymes of *S. latifolia* showed the lowest activities (Figure 9). On the contrary, the extracellular lignocellulose-degrading enzymes of *S. latifolia* were the most active in the PSM supplemented with 4 mM OA^-^. The trend of enzymes’ change in activity was consistent with lignocellulose degradation. Analysis of enzymatic activity demonstrated that the presence of excess OA inhibited the degradation of lignocellulose by reducing the activity of the extracellular lignocellulose-degrading enzymes secreted by *S. latifolia*.

## 4. Discussion

*Sparassis latifolia* is a widely cultivated fungal species in China, Japan, and South Korea. Recently, several extracts of this fungus have been described to show beneficial medicinal properties [29]. However, the slow mycelial propagation in solid media limits the potential for the commercial cultivation of the fungus. Therefore, there is a demand to explore novel strategies to shorten the growth cycle. However, the mechanisms underlying or affecting the growth rate of the mycelium during its saprophytic process are unclear.

OA plays an important role in fungal mycelia infection of the host plant by acting as a chelating agent to chelate with metal oxides or metal ions and form crystals in the extracellular mycelia [30]. In this study, following prolonged periods of cultivation on PDPA media, obvious crystalline material was observed around the mycelia of *S. latifolia*. These crystals were visible to the naked eye and were easily accessible for further observation (Figure 1). The crystalline components were identified as oxalate substances containing COM and COD (Figure 2). COM is the predominant form of calcium oxalate [31]. OA can chelate calcium ions present in pectin calcium. This process, in cooperation with enzymatic digestion, can accelerate the hydrolysis of the pectin layer, contributing to the degradation of lignocellulose and the colonization of the host [32,33]. Oxalate crystallization can also directly attack host tissues and weaken wood structure. Based on these observations, we expected that a large amount of OA would be formed during the growth of *S. latifolia*, which could combine with calcium ions in the culture medium to form oxalate crystals.

Many wood rot fungi have the ability to synthesize and secrete OA [34,35]. In the present study, the amount of OA secreted by the *S. latifolia* mycelium was measured. The results indicate that OA content increased significantly and accumulated continuously with the growth of mycelia. Further prolongation of the incubation (from day 10 to day 25) resulted in significantly increased content of OA (Figure 3). During infection, pathogenic fungi such as *Sclerotinia sclerotiorum* can secrete large amounts of OA to attack the defense system on the plant surface, making penetration easier. OA accumulates continuously at the infected sites, leading to its direct toxicity and causing cell necrosis in host plant tissues [36]. Therefore, the formation of OA may contribute to the infection of the host by *S. latifolia*, a facultative parasitic fungus.

A clear difference in the growth rate of mycelia could be observed in the presence of different concentrations of OA, with high concentrations of exogenous OA significantly inhibiting fungal growth (Figure 4). The accumulating OA reduces the pH of the culture medium, which eventually becomes no longer conducive to the optimal growth of mycelia [37]. However, in our experiments, a low concentration of exogenous OA (2.5 mM) improved the growth of *S. latifolia*. This OA concentration may represent ideal conditions, providing a suitable environment for wood decay [38]. The ability of fungi to degrade lignocellulose is due to their highly effective enzymatic systems. The ability of the pathogen to invade a host plant is closely correlated to the effectiveness of its lignocellulose degradation enzymes [39]. The concentration of OA has a significant effect on activity of these enzymes [40]. Various wood rot fungi are able to secrete OA, which can significantly promote the activity of lignocellulose-degrading enzymes [41]. Moreover, wood rot fungi have the ability to regulate the synthesis of OA, maintaining an optimal concentration [42]. However, in this study, the highest activity of lignocellulose-degrading enzymes was exhibited when the PSM was supplemented with an inhibitor of OA synthesis (4 mM OA^-^) (Figure 9). When the medium contained a high concentration of OA, the ability of *S. latifolia* to degrade pine sawdust decreased significantly. This observation is consistent with decreased activity of enzymes degrading lignocellulose (Figure 7 and Figure 8). We conclude that *S. latifolia* can secrete OA during its saprophytic process, resulting in the continuous increase in OA concentration. Based on the results presented, we feel confident in confirming that concentrations over 5 mM of OA inhibit the mycelial growth of *S. latifolia* and its wood decay enzymatic activity.

## 5. Conclusions

In summary, the present study confirmed that *S. latifolia* can secrete OA during its saprophytic process and chelate calcium ions in the environment to form calcium oxalate crystals. To our knowledge, this is the first study to report the relationship between the growth of *S. latifolia* and OA. Our observations provide new insight that high concentrations of OA may inhibit *S. latifolia*’s ability to grow saprophytically, reducing the mycelium growth rate. The findings provide the basis for further work aiming to improve the slow mycelial growth of *S. latifolia* and may prove helpful in improving the commercial cultivation of this fungus in future.

## Data Availability

All data presented in this study are available within this article.

## Abbreviations

OA, oxalic acid; COM, calcium oxalate monohydrate; COD, calcium oxalate dihydrate; XRD, X-ray diffraction; HPLC, high-performance liquid chromatography; PDPA, potato dextrose peptone agar; PDPB, potato dextrose peptone broth; PSM, sawdust medium; SEM, scanning electron microscope; TEM, transmission electron microscope; UV, ultraviolet; OA^-^, inhibitors of OA synthesis; MnP, manganese-dependent peroxidase; ABTS, 2,2′-azinodi-3-ethyl-benzothiazoline-6-sulfuric acid; CMCase, carboxymethyl cellulase; CK, normal medium or substrate.
